# Long-Term Asymmetric Hearing Affects Cochlear Implantation Outcomes Differently in Adults with Pre- and Postlingual Hearing Loss

**DOI:** 10.1371/journal.pone.0129167

**Published:** 2015-06-04

**Authors:** Isabelle Boisvert, Catherine M. McMahon, Richard C. Dowell, Björn Lyxell

**Affiliations:** 1 Department Linguistics, Macquarie University, Sydney, New South Wales, Australia; 2 HEARing Cooperative Research Centre, Melbourne, Victoria, Australia; 3 SCIC Cochlear Implant Program - An RIDBC service, Sydney, New South Wales, Australia; 4 Department of Audiology and Speech Pathology, The University of Melbourne, Melbourne, Victoria, Australia; 5 Audiology, Royal Victorian Eye and Ear Hospital, Melbourne, Victoria, Australia; 6 Department of Behavioural Sciences and Learning, Linköping University, Linköping, Sweden; 7 Linnaeus Centre HEAD, The Swedish Institute for Disability Research, Linköping, Sweden; Utrecht University, NETHERLANDS

## Abstract

In many countries, a single cochlear implant is offered as a treatment for a bilateral hearing loss. In cases where there is asymmetry in the amount of sound deprivation between the ears, there is a dilemma in choosing which ear should be implanted. In many clinics, the choice of ear has been guided by an assumption that the reorganisation of the auditory pathways caused by longer duration of deafness in one ear is associated with poorer implantation outcomes for that ear. This assumption, however, is mainly derived from studies of early childhood deafness. This study compared outcomes following implantation of the better or poorer ear in cases of long-term hearing asymmetries. Audiological records of 146 adults with bilateral hearing loss using a single hearing aid were reviewed. The unaided ear had 15 to 72 years of unaided severe to profound hearing loss before unilateral cochlear implantation. 98 received the implant in their long-term sound-deprived ear. A multiple regression analysis was conducted to assess the relative contribution of potential predictors to speech recognition performance after implantation. Duration of bilateral significant hearing loss and the presence of a prelingual hearing loss explained the majority of variance in speech recognition performance following cochlear implantation. For participants with postlingual hearing loss, similar outcomes were obtained by implanting either ear. With prelingual hearing loss, poorer outcomes were obtained when implanting the long-term sound-deprived ear, but the *duration* of the sound deprivation in the implanted ear did not reliably predict outcomes. Contrary to an apparent clinical consensus, duration of sound deprivation in one ear has limited value in predicting speech recognition outcomes of cochlear implantation in that ear. Outcomes of cochlear implantation are more closely related to the period of time for which the brain is deprived of auditory stimulation from both ears.

## Introduction

Cochlear implants have been dubbed ''the most successful of all neural prostheses'' [1], helping to partly restore hearing in more than 300 000 people around the world [2]. For most adults receiving a cochlear implant, only one ear is implanted despite a bilateral hearing loss. It is therefore desirable to choose the ear for implantation that optimises communication outcomes. The criteria for implantation are broadening and individuals with more residual hearing are being offered a cochlear implant as a solution to their communication difficulties. There is, however, a relative lack of evidence to guide clinicians in choosing the best ear for implantation. This is particularly the case for adult patients with asymmetric hearing thresholds or different durations of sound deprivation in each ear where they perceive one ear to be “better” and the other “poorer”. In these situations, clinicians and patients face the dilemma of either choosing to implant the poorer ear, where the outcomes are unknown, or risk implanting the better ear where residual hearing may be lost. Therefore this study specifically aimed to examine the effects of unilateral sound deprivation on cochlear implantation outcomes in patients with bilateral hearing loss.

Duration of deafness is commonly identified as one of the main predictors of implantation success in adults, where higher performance in speech recognition is associated with shorter durations of deafness [3–5]. The association between long duration of deafness and poor implantation outcomes is also supported by literature using animal models of deafness showing the degradation of peripheral neural structures [6], such as the spiral ganglion cells [7,8] and the cochlear nucleus [9]. These studies have been used to support the assumption that in humans, the longer an ear has been deprived of hearing the more limited the outcomes with the cochlear implant will be for that ear. This assumption, however, is largely derived from studies in which most participants had similar hearing thresholds and/or auditory stimulation in both ears [3,10,11], or in which a hearing aid (i.e. not a cochlear implant) was reintroduced after a period of sound deprivation [12]. This assumption is further reinforced by studies investigating outcomes of cochlear implantation and hearing asymmetries in early childhood deafness [13,14].

Conversely, in adults, better hearing in one ear could contribute to higher outcomes of implantation in the other ear [15]. This idea is derived from the suggestion that hearing in one ear may contribute to the preservation of: 1) peripheral neuronal structures of the other ear [16] and/or; 2) cognitive abilities related to speech processing such as phonological representations [17]. As indicated by Moore and Shannon [18]:

*“Optimization of the benefit from implants depend not only on the implant signal but also on its coupling to the central auditory system and other related brain systems to learn how to most efficiently obtain meaning from that signal”*.

Identifying whether the impact of duration of sound deprivation on cochlear implantation outcome is ear, or brain specific has considerable clinical implications. In particular, cochlear implant clinicians must regularly decide which ear to implant first [11,19–21]. It is often assumed that poorer outcomes will be obtained when implanting a long-term sound-deprived ear compared to an ear that continues to receive auditory stimulation [11,21,22]. Accordingly, Connell and Balkany (2006) recommends not to implant the deprived ear if that ear has >10 years of auditory deprivation. On the other hand, it is difficult for patients to sacrifice their only useful ear for cochlear implantation, even when hearing is limited. Further, an advantage of implanting the long-term sound-deprived ear is for patients to gain access to hearing from both ears by maintaining their hearing aid in their “better” ear [23–25].

This paper examines the impact of long-term unilateral sound deprivation on outcomes of implantation in individuals who had large differences in the duration of sound deprivation for each ear prior to implantation. This is done by comparing speech recognition outcomes in adults with bilateral hearing loss using a single hearing aid and implanted in either the aided (better) or long-term unaided (poorer) ear. The contribution of potential predictors to outcomes of cochlear implantation is also examined.

## Methods

### Study design and participants

Audiological data from adult participants (>18 years of age at implantation) with a bilateral hearing loss was included in this retrospective multicentre cohort study. All used only one hearing aid and had a severe (pure-tone threshold average_(500, 1k, 2kHz)_≥70dB) and unaided hearing loss in one ear for at least 15 years before unilateral cochlear implantation. Implantation was performed between 2000 and 2009. The surgery was conducted at one of the five following centres: the Québec Cochlear Implant Program (Canada), the Sydney Cochlear Implant Centre (Australia), the Melbourne Cochlear Implant Clinic (Australia), the Cochlear Implant Program at Linköping University Hospital (Sweden), and the Cochlear Implant Program at Karolinska University Hospital (Sweden). Participants were excluded from the study if there was evidence of surgical complication, device failure, or severe cognitive or psychiatric problems. A prelingual onset of hearing loss was defined as a severe degree of hearing loss before three years of age [26] in the sound-deprived ear.

146 participants were included in this study, 99 with a postlingual hearing loss. 98 of 146 (67%) received the cochlear implant in the sound-deprived (poorer) ear. Demographics are presented in Table 1.

**Table 1:** Demographics of participants.

		CI in better (aided) ear (n = 48)	CI in poorer (sound deprived) ear (n = 98)
Gender	Female (n)	25	51
	Male (n)	23	47
Onset	Prelingual (n)	19	28
	Postlingual (n)	29	70
Age (mean (SD)):		57.1y (17.8)	58.7y (17.3)
Duration of unilateral sound deprivation (mean (SD))		34.3y (14.1)	28.8y (11.5)
Duration of bilateral significant hearing loss (mean (SD))		7.9y (12.4)	8.3y (13.0)
Pure-tone threshold average—CI ear (mean (SD))		108.3dB (11.7)	113.1dB (10.1)
Pure-tone threshold average—Other ear (mean (SD))		112.6dB (10.4)	96.9dB (14.7)

CI: Cochlear implant.

The duration of bilateral significant hearing loss as defined in Boisvert et al. [15] was used to represent the duration for which an individual had very limited access to meaningful auditory input from either ear. Specifically, in the present study, the duration of bilateral significant hearing loss was the time for which two of the following factors were met in both ears, before implantation: i) the hearing loss was severe (pure-tone threshold average ≥70dBHL), ii) the use of the telephone was not possible, and/or iii) the speech recognition scores (SRSs) with optimally adjusted hearing aids were ≤30% for sentences or ≤10% for words. Information about telephone usage was based on patient report collected during the candidacy evaluation for cochlear implantation. The SRSs measured before and after implantation were obtained from the clinical files. These were measured with standardised audio-recorded material presented in free-field, at 60 or 65 dBSPL, based on the testing protocol of the respective centres. The SRS measured three to six months after the initial switch-on of the device were used as the functional outcome measure for this study. This was chosen to increase the sample size as it was the period where the largest amount of data was available across participating centres. To combine multilingual speech recognition data, the scores obtained in each country were standardised in relation to the mean score and standard deviation obtained by the population of adult cochlear implant recipients tested with the same tests during the same period of time. Standardised scores were then converted to percentile ranks and integrated.

Ethical review, guidance and approvals to conduct the study were obtained from Macquarie University, New South Wales Department of Health, and Royal Victorian Eye and Ear Hospital Human Research Ethics Committees in Australia. Written clinical consent was obtained to collect and analyse the data anonymously.

### Statistical analysis

The relative contribution of potential predictors to outcomes of implantation was assessed with a multiple regression analysis using the SRS obtained with the cochlear implant alone as the dependent variable. In a general linear model, the ear implanted (better or poorer) and the presence of a prelingual hearing loss were included as fixed factors, while age, duration of sound deprivation in the implanted ear, and duration of bilateral significant hearing loss were included as covariates. The frequency of use of bimodal hearing (cochlear implant in one ear and hearing aid in the other) by participants in each group was also examined. A mixed model analysis of variance was conducted to compare SRS obtained in the two groups when tested with the cochlear implant alone and in the everyday listening condition (i.e. with both the cochlear implant in one ear and the hearing aid in the other ear if the participant generally used this condition for listening at home). Comparisons were finally conducted separating participants with and without prelingual hearing loss.

## Results

### Predictors of speech recognition outcomes after implantation

Table 2 presents the relative contribution of the variables included in the multiple regression model. Duration of *bilateral* significant hearing loss and the presence of a prelingual hearing loss were found to explain the majority of variance in speech recognition scores following cochlear implantation (R^2^ = .26, F_(6,146)_ = 8.11, p<.001). Duration of sound deprivation in the implanted ear and placing the cochlear implant in the better or poorer ear did not significantly affect the outcomes.

**Table 2:** Variables included in the multiple regression to predict speech recognition performance after cochlear implantation.

Variables	B	*p*
Prelingual hearing loss	-31.7	<.001
Duration of bilateral significant hearing loss	-.51	.005
Age at implantation	-.42	.008
Interaction between prelingual loss and CI in better or poorer ear	-24.6	.011
^a^Duration of sound deprivation in the implanted ear	-.17	.49
^a^CI in better or poorer ear	-4.9	.58

CI: Cochlear implant.

^a^ These variables were not significant predictors in this model.

### Probability of successful use of hearing in both ears (bimodal hearing)

One argument often used to support implantation of the poorer ear is to increase patients’ probability of utilising bimodal hearing (i.e. using a hearing aid in one ear in combination with a cochlear implant in the other) [27]. Three to six months after implantation, only 8% of participants implanted in their better ear used bimodal hearing, in comparison to 72% of participants implanted in their poorer ear. Similar percentages were found when separating participants with pre- and postlingual hearing loss.

### Speech recognition outcomes with the cochlear implant in the better or poorer ear

A mixed model analysis of variance suggested no significant difference between the SRS obtained by participants implanted in their better ear (M = 47.1%, SD = 28.7) and participants implanted in their poorer ear (M = 40.0%, SD = 28.8; F_(1,127)_ = .54, p = .46). An interaction effect showed that the mean performance was improved for the group who received the cochlear implant in their poorer ear when tested in the everyday listening condition (M = 46.4%, SD = 27.6) compared to when tested with the cochlear implant alone (F_(1,127)_ = 9.64, p = .002). For participants who received the cochlear implant in their better ear, performance was similar with the cochlear implant alone and in the everyday listening condition (M = 46.7%, SD = 28.9). This is because many of the participants who received the implant in their poorer ear used a hearing aid in the non-implanted ear in combination with the cochlear implant.

**Fig 1:**
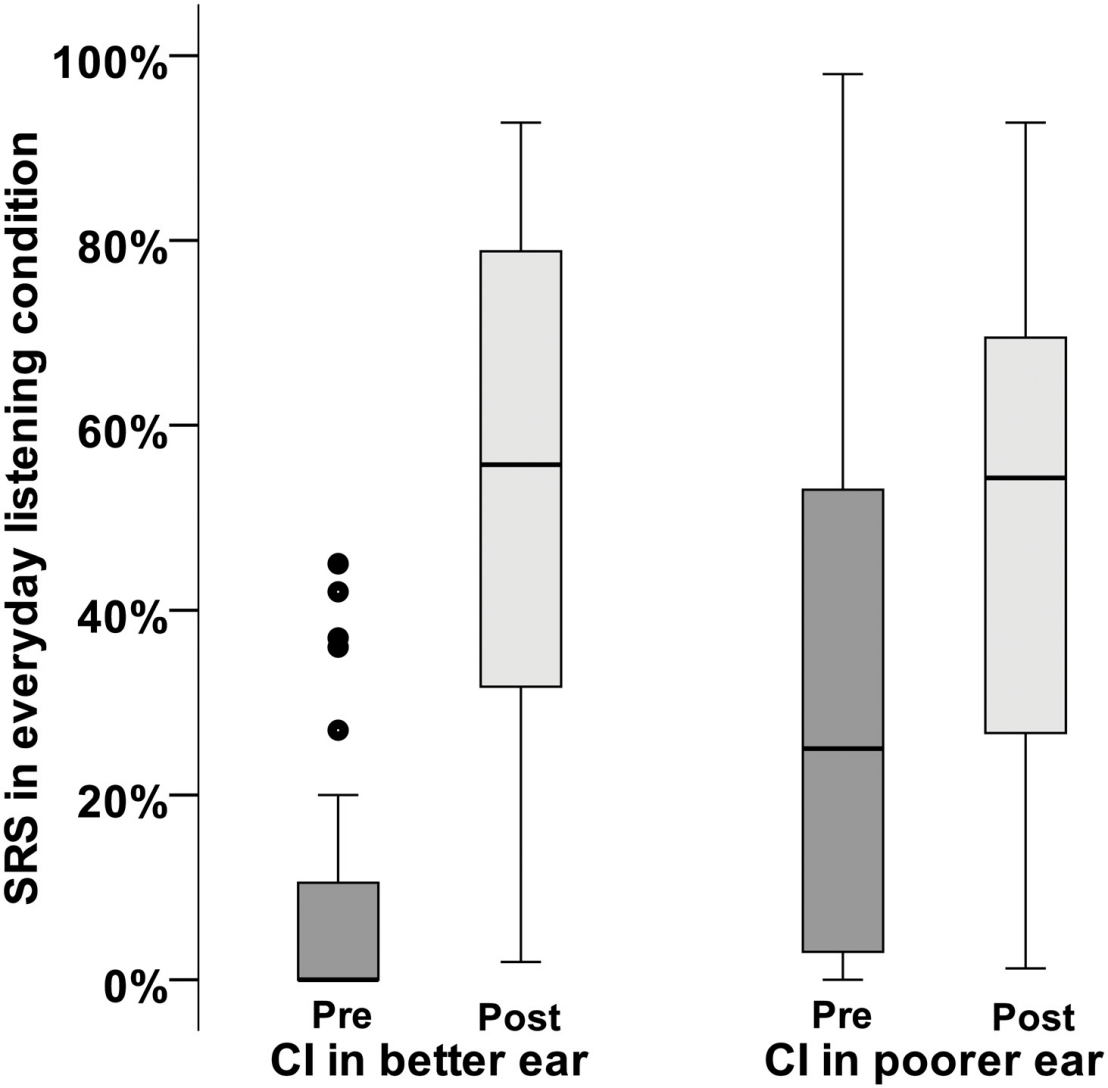
Speech recognition performance in the everyday listening condition measured before and after cochlear implantation in the better or poorer ear. Similar speech recognition performances were obtained after cochlear implantation of either a previously aided (better) or long-term sound-deprived (poorer) ear. The pre-implantation scores for the group implanted in their poorer ear were obtained with the non-implanted ear only (the poorer ear being unaided). CI: cochlear implant. The dots represent scores more than 1.5 interquartile range from the rest of the scores.

Although the speech recognition outcome specific to the implanted ear was similar between the groups after implantation, the overall improvement in SRS measured in the everyday listening condition pre- and post-implantation was greater for participants implanted in their better ear compared to participants implanted in the poorer ear (Fig 1). This is because those implanted in the poorer ear had greater SRS in their only hearing ear before implantation, which guided the choice of ear for implantation. Conversely, improvement in SRS specific to the implanted ear was greater for the group implanted in their poorer ear.

### Impact of pre- compared to postlingual hearing loss

**Fig 2:**
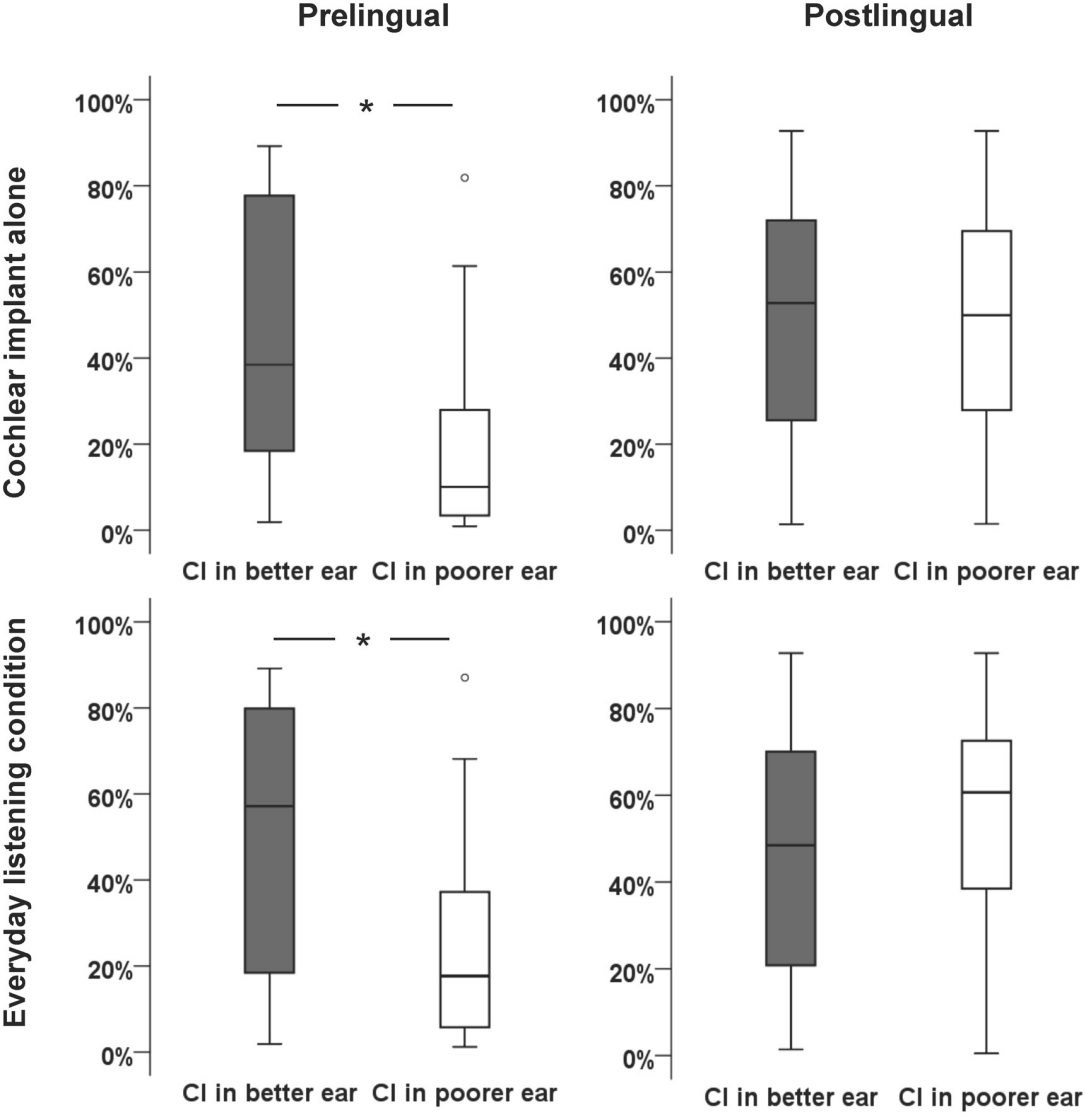
Outcome of cochlear implantation in adults with long-term hearing asymmetry. Similar speech recognition performances were obtained with the use of the cochlear implant alone and in the everyday listening condition for participants with postlingual hearing loss, implanted either in their previously aided (better) or long-term sound-deprived (poorer) ear. In adults with prelingual hearing loss, better performances were obtained with the cochlear implant placed in the better ear. *denotes a significant difference between groups (p<0.05). CI: cochlear implant.

Based on the results obtained with the multiple regression analysis, we separated outcomes of participants with postlingual and prelingual hearing loss (Table 3). An analysis of variance of the SRS when using the cochlear implant alone established significant variation between the groups (F_(3,142)_ = 8.53, p<.001). A post-hoc Tukey HSD test showed that participants with postlingual hearing loss obtain similar outcomes with the implant in the better (M = 47.6%, SD = 27.8), or poorer ear (M = 48.22%, SD = 27.22). The outcomes obtained by participants with postlingual hearing loss were also similar to those of participants with prelingual hearing loss implanted in their better ear (M = 46.5%, SD = 30.7), but not in their poorer ear (M = 19.3%, SD = 21.6; p≤.005). A similar pattern of results was found when considering the everyday listening condition. This result demonstrates that sound deprivation affects outcomes of implantation differently in adults with prelingual and postlingual hearing loss. Fig 2 presents the SRS obtained by participants with pre- and postlingual hearing loss, implanted in their better or poorer ear.

**Table 3:** Age and hearing loss characteristics of participants with pre- and postlingual hearing loss.

	Prelingual		Postlingual	
	(mean (SD))		(mean (SD))	
Ear implanted	Better	Poorer	Better	Poorer
	n = 19	n = 28	n = 29	n = 70
Age (y)	44.6	42.8	65.2	65.1
	(15.0)	(15.6)	(14.6)	(13.4)
Duration of unilateral sound deprivation (y)	36.5	32.3	32.8	27.4
	(13.9)	(13.8)	(14.3)	(10.3)
Duration of bilateral significant hearing loss (y)	11.2	16.5	5.7	5.0
	(17.1)	(17.7)	(7.5)	(8.8)
Pure-tone threshold average—CI ear (dB)	110.4	111.7	106.9	113.7
	(10.3)	(11.3)	(12.6)	(9.7)
Aided speech recognition score pre-CI* (%)	9.7	22.6	6.5	39.8
	(15.0)	(27.4)	(11.9)	(30.9)

CI: Cochlear implant

*Score obtained with the better hearing ear only (the poorer ear being unaided)

## Discussion

The data suggest that in adults with postlingual hearing loss, contrary to an apparent clinical consensus [21,22], a long duration of sound deprivation in one ear has little influence on speech recognition outcomes after cochlear implantation in that ear. In adults with 15 to 66 years of postlingual unilateral sound deprivation, similar speech recognition outcomes were obtained with the implant placed in the better (previously aided) or the poorer (long-term sound-deprived) ear.

The majority of individuals who received a cochlear implant in their poorer ear continued using a hearing aid in the opposite ear, enabling the additional benefits of hearing with two ears. In comparison, only a minor proportion of adults implanted in their better ear gained access to binaural hearing after implantation. Several studies have demonstrated consistent benefits of bimodal hearing over a single cochlear implant used alone [28–30], in particular for listening in noisy environments.

The presence of a prelingual hearing loss in the implanted ear was identified as a main predictor of cochlear implantation outcomes. Consistent with previous studies ([31–33], see [34] for a review, [35]), significantly poorer outcomes were obtained with the cochlear implant placed in adult ears deafened at birth or in early childhood. In addition, an interaction effect was found between a prelingual hearing loss and the choice of ear for implantation. That is, the *presence of* a long duration unilateral sound deprivation appears to negatively impact on implantation in a prelingually, but not a postlingually, deafened ear. This finding suggests that auditory deprivation in the first few years of life has a different effect to auditory deprivation after the acquisition of higher level auditory and language skills. This is consistent with studies that identify a critical period for the development of auditory skills and spoken language between birth and four years [36,37]. It is also consistent with the findings of Gordon et al. [13] and of Kral et al. [14] suggesting that auditory asymmetry in early childhood is associated with a reorganisation of the bilateral auditory pathways. This reorganisation occuring in early childhood is then associated to different outcomes of implantation whether the hearing impaired ear was aided or unaided for a long duration (≥15 years) before cochlear implantation.

Importantly, for adults with postlingual deafness, duration of *bilateral* significant hearing loss was identified as a significant predictor of cochlear implantation outcomes, in contrast to the duration of unilateral sound deprivation, or whether the cochlear implant was placed in the better or poorer ear. This finding supports the notion that, for adults with postlingual deafness, outcomes of cochlear implantation are more closely related to the total auditory stimulation an individual has been exposed to, coming from either ear, and not from one specific ear [4,38,39].

The results of the current study confirm and extend previous findings supporting the relationship between outcomes of implantation and the total auditory receptivity of the brain as opposed to ear-specific events [4,38–41]. Most notably, Francis et al. [40] examined speech recognition scores of adults who had a severe hearing loss in one ear and a profound hearing loss in the other. All received a cochlear implant in their profoundly deaf ear. They found that adults with asymmetric hearing thresholds implanted in their profoundly deaf ear perform similarly to adults with a cochlear implant who had a severe hearing loss in both ears, and better than adults with a profound hearing loss in both ears [40]. Another example is the study by Freidland et al. [4], which showed that a formula predicting implantation outcomes developed with patients who received an implant in their better ear could reliably predict outcomes in patients implanted in their poorer ear. Their results further support that outcomes of implantation in postlingually deafened adults are weakly, if at all, related to the choice of ear for implantation. The methodology used in the present study is unique as it ensured there was a considerable difference in auditory experience between the ears, increasing the likelihood of observing an ear-specific effect if it was present. No such effect was observed with adults with a postlingual hearing loss.

Taken together, these results lead to a considerable shift in perspective when discussing outcomes of cochlear implantation. In adults, duration of sound deprivation in one ear has limited value to predict implantation outcomes in that ear. In cases of postlingual deafness, similar outcomes can be obtained by implanting the better or poorer ear. In cases of prelingual deafness, although poorer outcomes are obtained when implanting the poorer, long-term sound-deprived ear, the *duration* of sound deprivation of the implanted ear cannot reliably predict outcomes. Further neurophysiological studies are needed to understand the underlying mechanisms allowing such outcomes.

## Supporting Information

S1 File The supporting information file contains all de-identified data that has been used for the analyses presented in this article. (XLSX)
